# Identification and validation of the methylation biomarkers of non-small cell lung cancer (NSCLC)

**DOI:** 10.1186/s13148-014-0035-3

**Published:** 2015-01-22

**Authors:** Shicheng Guo, Fengyang Yan, Jibin Xu, Yang Bao, Ji Zhu, Xiaotian Wang, Junjie Wu, Yi Li, Weilin Pu, Yan Liu, Zhengwen Jiang, Yanyun Ma, Xiaofeng Chen, Momiao Xiong, Li Jin, Jiucun Wang

**Affiliations:** State Key Laboratory of Genetic Engineering and Ministry of Education Key Laboratory of Contemporary Anthropology, Collaborative Innovation Center for Genetics and Development, School of Life Sciences and Institutes of Biomedical Sciences, Fudan University Jiangwan Campus, 2005 Songhu Road, Shanghai, 200438 China; Department of Cardiothoracic Surgery, Changzheng Hospital of Shanghai, Fengyang Road 415, Shanghai, 200000 China; Yangzhou No.1 People’s Hospital, 368 Hanjiang Road, Yangzhou, 225001 China; Department of Cardiothoracic Surgery, Changhai Hospital of Shanghai, Changhai Road 168, Shanghai, 200433 China; Department of Pneumology, Changhai Hospital of Shanghai, Changhai Road 168, Shanghai, 200433 China; Center for Genetic & Genomic Analysis, Genesky Biotechnologies Inc., 787 Kangqiao Road, Shanghai, 201203 China; Department of Cardiothoracic Surgery, Huashan Hospital, Fudan University, 12 Wulumuqi Road, Shanghai, 200040 China; Human Genetics Center, The University of Texas School of Public Health, 1200 Herman Pressler, Houston, Texas 77030 USA; Fudan-Taizhou Institute of Health Sciences, 1 Yaocheng Road, Taizhou, Jiangsu 225300 China

**Keywords:** Non-small cell lung cancer, DNA methylation, Biomarker, Batch effect elimination, Diagnosis

## Abstract

**Background:**

DNA methylation was suggested as the promising biomarker for lung cancer diagnosis. However, it is a great challenge to search for the optimal combination of methylation biomarkers to obtain maximum diagnostic performance.

**Results:**

In this study, we developed a panel of DNA methylation biomarkers and validated their diagnostic efficiency for non-small cell lung cancer (NSCLC) in a large Chinese Han NSCLC retrospective cohort. Three high-throughput DNA methylation microarray datasets (458 samples) were collected in the discovery stage. After normalization, batch effect elimination and integration, significantly differentially methylated genes and the best combination of the biomarkers were determined by the leave-one-out SVM (support vector machine) feature selection procedure. Then, candidate promoters were examined by the methylation status determined single nucleotide primer extension technique (MSD-SNuPET) in an independent set of 150 pairwise NSCLC/normal tissues. Four statistical models with fivefold cross-validation were used to evaluate the performance of the discriminatory algorithms. The sensitivity, specificity and accuracy were 86.3%, 95.7% and 91%, respectively, in Bayes tree model. The logistic regression model incorporated five gene methylation signatures at AGTR1, GALR1, SLC5A8, ZMYND10 and NTSR1, adjusted for age, sex and smoking, showed robust performances in which the sensitivity, specificity, accuracy, and area under the curve (AUC) were 78%, 97%, 87%, and 0.91, respectively.

**Conclusions:**

In summary, a high-throughput DNA methylation microarray dataset followed by batch effect elimination can be a good strategy to discover optimal DNA methylation diagnostic panels. Methylation profiles of AGTR1, GALR1, SLC5A8, ZMYND10 and NTSR1, could be an effective methylation-based assay for NSCLC diagnosis.

**Electronic supplementary material:**

The online version of this article (doi:10.1186/s13148-014-0035-3) contains supplementary material, which is available to authorized users.

## Background

Lung cancer, a complex disease involving both genetic and epigenetic changes, is the leading cause of cancer deaths worldwide [1]. About 80% of primary lung cancers are non-small cell lung carcinoma (NSCLC), which is characterized by a long asymptomatic latency and poor prognosis. While the overall 5-year survival rates for late stage III and IV of NSCLC patients were just 5% to 14% and 1%, respectively, the rate could increase to 50% for the early stage of the NSCLC patients who are typically treated with surgery [2]. Many imaging and cytology-based strategies have been employed in NSCLC diagnosis; however, none of them have yet been proven completely effective in reducing the mortality. The advances in molecular profiling of NSCLC over the past decade have made a paradigm shift in its diagnosis and treatment.

Among all the genetic variations, single nucleotides polymorphisms (SNPs) have been considered as the most stable biomarker for heritable disease, since the status of the SNPs can be detected with almost 100% accuracy and unchanged during the entire life. It is specific and powerful for a single gene-caused disease. However, for complex diseases, such as cancers, the prediction power of SNPs is limited. A plethora of studies have shown that AUCs of the prediction model based on significant SNPs can confer only 0.54 to 0.55 for non-small cell lung cancer [3] and 0.54 to 0.60 for thyroid cancer [4], which has been considered as one of highest familial-risk carcinomas among all kinds of cancers. Molecular biomarkers such as mRNA, microRNA and protein for NSCLC diagnosis have been developed and investigated in the past decades. However, their accuracy for diagnosis of NSCLC is far from reaching clinical implementation, in which >90% sensitivity and specificity of diagnosis should be guaranteed.

DNA methylation, which is one of the most important mechanisms involved in gene and microRNA expression regulation [5] and in alternative gene splicing [6], plays important roles in the early stage of cancer. Because it is stable and easily detected qualitatively or quantitatively, DNA methylation was taken as the most promising diagnostic marker for the early detection of cancer [7,8] when compared with SNP/mutation [4], copy number variations (CNVs) [9] and gene/microRNA expression [10]. Hundreds of aberrant DNA methylation changes in the early stage of NSCLC have been identified in the past decades [11,12]. However, despite several diagnostic panels having been developed [13], these studies on DNA methylation in NSCLC were still limited by their small sample size, low number of selected genes and qualitative rather than quantitative DNA methylation. These limitations would cause low reproducibility of the assay and explain why the majority of these studies could not be replicated.

In our previous study, we found that prediction ability is limited when the prediction model only includes the methylation status of a single gene, even for a classic tumor suppressor gene [14]. A diagnostic panel with several genes would be a promising approach to achieve better prediction performance for clinical utility. Methylation microarrays measure the methylation levels of thousands of genes in a single assay. These arrays are a revolutionary tool for identifying genes whose methylation changes in response to a specific situation, such as different development stages, physiological status or pathological status, and provide fundamental data for feature selection to construct the best combination of the predictive variables. In addition, a large number of public methylation microarray datasets have been shared in certain database, such as Gene Expression Omnibus (GEO). The stability and reproducibility of the prediction model would be significantly increased when multiple datasets with the same study design are pooled together. However, methylation array results can be greatly affected by a variety of nonbiological variables, such as methods for DNA isolation, bisulfite conversion, probe processing and scanning, reagents from different companies, different technicians or even different atmospheric ozone levels. Usually, the term ‘batch’ refers to microarrays processed at one site over a short period of time using the same platform. The cumulative error introduced by these time, place and situation-dependent variations is referred to as batch effects. In terms of different study, the methylation microarray data were created in different times, places, and by different technicians and so on; therefore, the main variation among data would be shown as a batch effect. In our previous study, we found that the ComBat algorithm could remove such noise (batch signal or each individual study) from the dataset with powerful efficiency when adjusted with additional and multiple effects of the batch information [15,16], which provide the prerequisite to combine the methylation array dataset to increase the sample size of the statistical analysis.

In the present study, we first systematically integrated three independent high-throughput DNA methylation datasets from the GEO [17] and TCGA projects (Additional file 1: Table S1). An optimized DNA methylation combination was established through the feature selection procedure after preliminary normalization and batch effect elimination with the ComBat algorithm among the datasets to maximize the NSCLC prediction performance. Methylation statuses for five genes - *AGTR1*, *GALR1*, SLC5A8, *ZMYND10* and *NTSR1-* were identified as being the most powerful combination for the NSCLC prediction. Then, to further evaluate their performance for diagnosis, we designed a novel methylation status as determined by the single nucleotide primer extension technique (MSD-SNuPET) for the simultaneous quantification of methylation at these five methylated loci. These five significantly differentially methylated genes were used to validate the results in 150 pairs of NSCLC and normal tissues from a Chinese Han population with MSD-SNuPET.

## Results

### Public dataset collection, batch effect elimination and candidate gene selection

**Figure 1:**
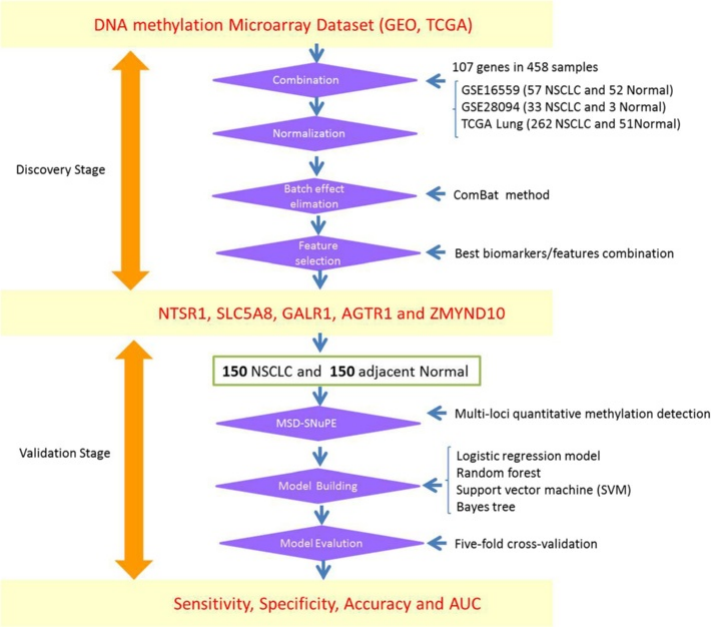
**Sketch of the study design and pipeline.** Candidate biomarkers were selected from meta-analysis to multiple high-throughput DNA methylation microarrays. The significant or best feature combination was screened in an independent validation study of non-small cell lung cancer (NSCLC) with the methylation status determined single nucleotide primer extension technique (MSD-SNuPET) technique.

**Figure 2:**
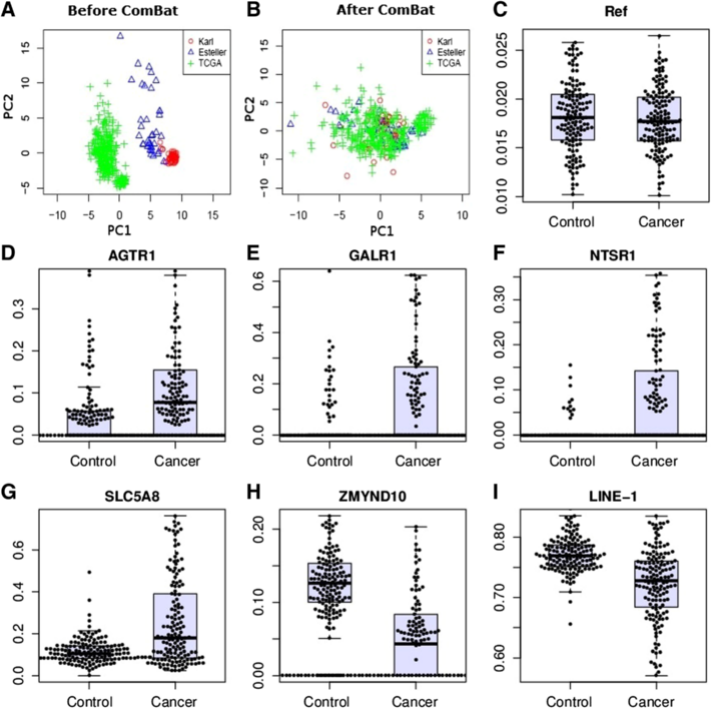
***ComBat*** **treatment and methylation status determined single nucleotide primer extension technique (MSD-SNuPET).** Principal component analysis was applied to show the efficiency of the elimination of *ComBat*. **A**, **B**, A total of 120 probe sets with DNA methylation values after background and quantile normalization in a set of 352 non-small cell lung cancer (NSCLC) and 106 normal samples. X and Y axes represent the first and second principal components (PC1 and PC2), respectively. **C-I** were validation of the methylation status of the five candidate markers in an independent samples. Y-axis represents absolute DNA methylation percentage from MSD- SNuPET. *LINE-1* and Reference were taken as the positive and negative control for MSD- SNuPET.

NSCLC-related public DNA methylation microarrays were searched through the Gene Expression Omnibus (GEO), ArrayExpress and TCGA projects. In total, three independent NSCLC datasets were created with a total of 458 microarrays, which included 352 NSCLC and 106 normal tissues (Figure 1 and Additional file 1: Table S1). A batch effect significantly existed among the datasets, and this was shown in the first and second principle components. We observed that the samples were clustered mainly by studies rather than by tumor and normal tissue samples (Figure 2A). *ComBat*, an empirical Bayes method, was used to eliminate the batch effects after quantile normalization in the three datasets. As a result, the batch effect was largely removed by *ComBat* (Figure 2B). In addition, as the hierarchical cluster analysis showed, biological information was highly preserved after batch effect elimination (Additional file 1: Figure S2). The SVM was used to conduct feature selection and assess the prediction abilities with leaving-one-out cross-validation. The accuracy of the SVM for classifying NSCLC was 98.98%, in the test set. Among the 112 shared probes, five CpG sites (*NTSR1*, *SLC5A8*, *GALR1*, *AGTR1* and *ZMYND10*) were selected in the feature selection stage. We found these five genes were significantly differentially methylated between the tumor and normal tissue samples. In detail, meta-analysis of the DNA methylation microarrays showed that *NTSR1* (*P* = 5.4 × 10^-15^), *SLC5A8* (*P* = 5.9 × 10^-9^), *GALR1* (*P* = 9.9 × 10^-10^) and *AGTR1* (*P* = 6.7 × 10^-5^) were significantly hypermethylated in NSCLC, whereas *ZMYND10* (*P* = 6.2 × 10^-20^) was significantly hypomethylated in NSCLC (Additional file 1: Figure S3). These results suggested that the selected five predictors would be potential biomarkers for the NSCLC diagnosis. To further evaluate their performance for diagnosis of NSCLC, we developed a panel of these five DNA methylation biomarkers and validated their diagnostic efficiency in 150 paired NSCLC and normal tissue samples in China.

### Methylation status validation with methylation status determined single nucleotide primer extension technique

In order to validate the results from the meta-analysis, methylation status of the above five genes were detected with MSD-SNuPET in 150 pairs of NSCLC and adjacent normal tissues. The characteristics of patients were showed in Table 1. Consistent with the microarray data, the absolute DNA methylation percentage of these five genes were significantly differentially methylated between NSCLC and normal tissues (Table 2, Figure 2C-I). Logistic regression analysis showed that hypermethylated *NTSR1*, *SLC5A8*, *GALR1*, and *AGTR1* and hypomethylated *ZMYND10* were significantly associated with the NSCLC when risk-adjusted for age, sex and smoking status with the *P* value of 5.9 × 10^-7^, 7.8 × 10^-9^, 2.3 × 10^-6^, 1.3 × 10^-6^, and 5.2 × 10^-8^, respectively (Table 2). The *MSD*-SNuPET results showed that the methylation of *LINE-1* was significantly lower in NSCLC than normal tissue (*t*-test, *P* = 2.39 × 10^-12^). Additionally, DNA methylation of *LINE-1* was significantly associated with sex (R^2^ = 0.18, *P* value = 0.0087), which was highly consistent with the previous reports about the methylation status of this gene [18,19] and supports the high credibility of the MSD-SNuPET. The prediction ability for each gene separately was also evaluated by logistic regression. Moderate prediction ability was identified, in which sensitivity ranges from 44.3% to 73.15%, specificity ranges from 79.59% to 94.56%, and AUC ranges from 0.67 to 0.80 (Table 2) were demonstrated. Correlation analysis showed that there was no co-methylation among the five genes. In addition, no significant association was observed between any of the five genes with age, smoking, TNM stage, lung cancer differentiation and lung cancer subtype (Ad or Sc) in both the univariate and multivariate association models in our study. However, a significant association between sex and *SLC5A8* (*P* = 0.0001), *ZMYND10* (*P* = 0.045) was identified, which might indicate a specific biological mechanism of *SLC5A8* and *ZMYND10* in the tumorigenesis of NSCLC. Protein-protein interaction networks from *String 9.0* showed that there were comprehensive networks for both *NTSR1* and *GALR1*. The majority of these genes were cancer-related genes, which have been reported to play important roles in cancer initiation, progress or therapy, such as *S100A9*, *NGF*, *TAC1*, *CCK*, *FPR2*, *ADRA1B*, and *CCL21* in the gene-gene interaction networks (Additional file 1: Figure S4).

**Table 1:** **Characteristics of patients**

	**NSCLC = 150**
Age	40 (IQR = 15 to 65)
Sex	
Male	120
Female	30
Smoke Status^a^	
Non-smokers (never)	41
Smokers (ever)	96
Histology	
Adenocarcinoma	53
Squamous cell carcinoma	63
Others^b^	34
Stage^c^	
I (IA,IB)	42 (10,32)
II (IIA,IIB)	48 (16,32)
III (IIIA,IIIB)	46 (41,5)
IV	2
Differentiation^d^	
Well/Moderate	74
Poor	30

NSCLC, non-small cell lung cancer; ^a^Smokers include former and current smoker individuals. ^b^Others include adenosquamous carcinoma (ADSQ), bronchioloalveolar carcinoma, mucoepidermoid lung tumor, Sarcomatoid carcinoma. ^c^TNM Stages were assessed by the seventh edition of TNM classification criteria. ^d^Qualitative assessment of tumor differentiation was based on sum of the architecture score and cytologic atypia score (2 = well differentiated, 3 = moderately differentiated, 4 = poorly differentiated).

**Table 2:** **Differential methylation in non-small cell lung cancers (NSCLCs)**

	**AMP** ^**a**^ **(NSCLC)**	**AMP (Control)**	***P*** **value** ^**b**^	**log**_**10**_ **(OR) (95% CI)**	***P*** **value** ^**c**^	**Sen** ^**d**^	**Spe** ^**d**^	**AUC** ^**d**^
*AGTR1*	12.88%	4.48%	1.06 × 10^-7^	3.49 (2.08, 4.91)	1.30 × 10^-6^	59.73%	79.59%	0.71
*GALR1*	18.31%	2.91%	6.58 × 10^-9^	2.56 (1.5, 3.63)	2.30 × 10^-6^	46.98%	85.03%	0.67
*NTSR1*	9.37%	0.56%	1.09 × 10^-9^	9.02 (5.48, 12.55)	5.90 × 10^-7^	44.30%	94.56%	0.70
*SLC5A8*	25.59%	11.66%	4.77 × 10^-12^	3.80 (2.51, 5.09)	7.80 × 10^-9^	52.35%	88.44%	0.67
*ZMYND10*	6.95%	12.82%	1.08 × 10^-7^	-4.61 (-6.27, -2.95)	5.20 × 10^-8^	73.15%	92.52%	0.80
*LINE-1*	72.10%	76.76%	2.39 × 10^-12^	-10.3 (-13.5, -7.2)	1.80 × 10^-10^	-	-	-
Reference^e^	1.78%	1.83%	2.85 × 10^-1^	-19.37 (-45.35, 6.62)	0.14	-	-	-

^a^Differential methylation analysis was conducted between 150 NSCLC and adjacent normal tissues. AMP represents average methylation percentage. ^b^ *P* value^b^ is the Bonferroni adjusted *P* value which is based on paired *t*-test comparing the intensity of the methylation signals between case and control. ^c^The log_10_(OR) and *P* value^c^ represent log-transformed odds ratio and *P* value based on logistic regression adjusted by sex, age and smoking status. ^d^Sensitivity, specificity and area under the curve (AUC) were calculated with a logistic regression prediction model without adjustment for sex, age and smoking status. ^e^Reference site was a C site that was not in the CpG site; therefore, no or a low-methylated signal would be detected and a nonsignificant association should be detected between cancer and normal tissues.

### Sensitivity, specificity and accuracy of the diagnosis panel

Several classification methods, including logistic regression model, random forest, support vector machine (SVM), and Bayes tree, were used to construct effective diagnosis models for cancer prediction based on MSD-SNuPET results. No significant unbalances were found in the train and test dataset, which suggested the prediction models were credible and stable. Fivefold cross validation was used to evaluate the performance of the classifiers. As a result, the Bayes tree was the most powerful model for the diagnosis of NSCLC, whose sensitivity (Sen), specificity (Spe) and classification accuracy (Acc) were 86%, 96% and 91% (Table 3), respectively. Other classification methods had similar performance, and the worst classifier was the logistic regression. However, even the logistic regression model incorporated the same five genes mentioned above, and in this model, the sensitivity, specificity, classification accuracy, and area under the curve (AUC) could reach 78%, 97%, 87%, and 0.906 (95% CI: 0.89 to 0.91), respectively, after being adjusted for age, sex and smoking. The logistic regression still showed the potential diagnostic significance of the five methylated genes. In addition, prediction abilities between smoking and non-smoking, adenocarcinoma and squamous cell carcinoma, early stage (I and II) and late stage (III and IV), and well or moderately and poorly differentiated populations were assessed under the Bayes tree model. We found there is no significant differential performance between smoking (Acc = 92.1%, 95% CI: 90.6% to 93.6% ) and non-smoking (Acc = 0.939, 95% CI: 0.935 to 0.943), adenocarcinoma (Acc = 0.82, 95% CI: 0.72 to 0.92) and squamous cell carcinoma (Acc = 0.94, 95% CI: 0.87 to 0.95), early stage (Acc = 0.87, 95% CI: 0.75 to 0.87) and late stage (Acc = 0.92, 95% CI: 0.82 to 0.92), while a significant difference (permutation test, *P* <10 to 10) was found between well or moderately (Acc = 0.9, 95% CI: 0.83 to 0.91) and poorly differentiated populations (Acc = 0.73, 95% CI: 0.5 to 0.74), which suggested further research should be considered.

**Table 3:** **Diagnosis accuracy, sensitivity and specificity based on several classification methods with fivefold cross-validation**

	**Test**			**Train**		
	**Sensitivity**	**Specificity**	**Accuracy**	**Sensitivity**	**Specificity**	**Accuracy**
Logistic regression	0.791	0.993	0.891	0.775	0.969	0.871
SVM^a^	0.897	0.977	0.937	0.855	0.941	0.897
Random forest	0.934	0.928	0.931	0.890	0.886	0.886
Bayes tree	0.911	0.976	0.944	0.863	0.957	0.909

^a^SVM represents support vector machines and Kernel Methods. Sensitivity, specificity and classification accuracy were its mean value in fivefold validations with 1,000 replications. In the main body of the manuscript, sensitivity, specificity and accuracy were derived from training result of the classification.

## Discussion

NSCLC early diagnosis and corresponding surgical intervention are taken as the most effective methods for increasing the survival time and for decreasing mortality from NSCLC death. Since the global change of DNA methylation occurred in the beginning of the carcinogenesis, DNA methylation has been considered as the most powerful biomarker for early detection, even screening [20]. In the present study, the two stage biomarker discovery pipeline was applied to optimize the combination of DNA methylation biomarkers for NSCLC diagnosis. The optimal biomarker combination was identified using 107 genes in a large discovery dataset. A novel DNA methylation diagnosis panel of five genes (*NTSR1*, *SLC5A8*, *GALR1*, *AGTR1* and *ZMYND10*) was identified. The DNA methylation diagnosis panel was then validated in another independent NSCLC study. A multi-loci DNA methylation detection method (MSD-SNuPET), was conducted to determine the absolute quantitative methylation level of the five genes in 150 pairs of NSCLC and adjacent normal tissues from a Chinese Han population. In the validation stage, the Bayes tree model shows the highest sensitivity, specificity and accuracy for NSCLC diagnosis based on the five genes, which is potential for clinical application.

It is important that five candidate biomarkers have been investigated widely in cancer research. Neurotensin receptor-1 (*NTSR1*) is a G-protein coupled receptor (*GPCR*). It has been widely reported to be associated with carcinogenesis, cancer progression [21] and prognosis [22,23]. Previous evidence showed the potential use of the *NTSR1* as a biomarker for cancer progression and as a component of personalized medicine in selective cancers [24], and this is consistent with our present result. *GALR1*, galanin receptor subtype 2, suppresses cell proliferation in several cancers such as head and neck [25,26] and oral squamous cell carcinoma [27]. Gene expression inactivation of *GALR1* can be caused by promoter hypermethylation [25]. Meanwhile, *GALR1* has also been a subtype determining gene in breast cancer, which suggests its potentially powerful role in cancer diagnosis. *SLC5A8* (solute carrier family 5, member 8) is a tumor suppressor gene and is usually suppressed in colon, and gastric cancers [28-30]. *ZMYND10* (Zinc finger, MYND-type containing 10) has recently been identified as a candidate tumor suppressor gene due to the occurrence of mis-sense mutations and loss of its expression in lung cancer.

Multicellular tissue is a great challenge in epigenetic studies. On one side, cancer tissues include cancer cells (epithelial cells), mesenchymal cells and so on. However, the proportion (at least 70% in general) of the tumor cells in cancer tissue is always significantly much higher than that of other cells. On the other side, normal tissues also include epithelial cells, mesenchymal cells and some others. In the present study, the null hypothesis is that the methylation level in the cancer tissue (mixed cells) is the same with normal tissue (mixed cells). The alternative hypothesis is that the methylation level in the cancer tissue (mixed cells) is different from normal tissue (mixed cells). We used the paired *t*-test to test the difference in the mean of the methylation between cancer tissue and normal tissue. The background or the noises from the adjacent non-cancer cells could be adjusted from the cancer cells when the methylation profiles of the corresponding cells were established.

All the results in the present study were based on quantitative signals of the DNA methylation. We also conducted analyses that were based on discrete DNA methylation signals in which beta values <0.2 were defined as the un-methylated CpGs; beta values >0.8 were defined as the full methylated CpGs, and beta values between 0.2 and 0.8 define semi-methylated CpGs. In this condition, five genes were still significantly differentially methylated between the NSCLC and normal tissues. No significant changes were found in classification sensitivity, specificity and accuracy. Also, the sensitivity, specificity and AUC of diagnosis with one gene added to the model each time are summarized in the Additional file 1: Figure S5; in these cases, we found that sensitivity and AUC gradually increased, step by step.

Lung cancer diagnosis is a challenging problem. In order to discover a potential panel of DNA methylation-based biomarkers for diagnosis of NSCLC, we should perform a genome-wide search for an optional combination of tens or hundreds of loci from the genome-wide DNA methylation profile. Integration analysis of interplatform, genome-wide DNA methylation datasets with appreciated data normalization and batch effect elimination could provide optimal biomarker combinations in a large sample population to obtain maximum diagnosis efficiency. With this approach, we identified a five-gene signature including *AGTR1*, *GALR1*, *SLC5A8*, *ZMYND10* and *NTSR1*, which could provide high diagnostic sensitivity and specificity.

## Conclusions

Integrated analysis of multiple-platform high-throughput DNA methylation microarray datasets followed by batch effect elimination is a good approach to discover diagnostic biomarker panels for NSCLC. Methylation profiles of *AGTR1*, *GALR1*, *SLC5A8*, *ZMYND10* and *NTSR1* would be an effective methylation-based assay for the NSCLC diagnosis.

## Methods

### Study design and pipeline description

Public high-throughput microarray databases that include GEO and ArrayExpress were searched to collect NSCLC-related DNA methylation microarray data. Non-small cell lung cancer and/or methylation were taken as the key words in the retrieval procedure. Although a large number studies have been conducted in NSCLC biomarker research, only two GSE records were retrieved, including GSE16559 and GSE28094. GSE16559, which included 57 NSCLC and 52 normal tissue samples, was used to discover aberrant DNA methylation in lung adenocarcinoma and mesothelioma. GSE28094, with 33 NSCLC and 3 normal tissue samples, was designed to make the DNA methylation fingerprint with 1,628 human samples of different tissues and statuses. Both of these two datasets were based on the *Illumina GoldenGate* platform, which includes 371 genes with 1,536 loci. Additionally, the CGA project is another comprehensive study that included 262 NSCLC and 51 normal tissue samples. Infinium methylation 27 K with 14,495 genes and 27,578 loci were used to perform the DNA methylation profiling. The number of DNA methylation genes shared by these two methylation microarray platforms was 107 genes (112 probes). Eventually, DNA methylation profiling data of 458 NSCLC-associated samples (352 NSCLC and 106 normal tissue) were obtained from the above three public datasets. These data will be taken as the primary data in the biomarker discovery stage (Additional file 1: Table S1).

When the microarray is provided as fluorescent signals, the gene methylation level was calculated with the fluorescent signals of methylation and un-methylation alleles by the traditional function of

$$ \mathrm{beta}=\frac{max\left(\mathrm{M},0\right)}{max\left(\mathrm{M},0\right)+ max\left(\mathrm{U},0\right)}. $$

where M and U represent the signal intensities for about 30 methylated (M) and un-methylated (U) probes on the array. Background-correction was conducted according to the recommended methods for each platform. K-nearest neighbor imputation (KNN imputation) was performed to deal with the missing values. A total of 112 probes were shared between these two microarray platforms. DNA methylation signals of these probes were combined for all the samples. Quantile normalization was applied to combine all the data from different studies. To further reduce biases, we use the batch effect elimination tool, *ComBat*, to eliminate the batch effects that exist in independent datasets [15]. In the present study, we use the principal component analysis (PCA) to visualize the extension of the elimination of batch effect by observing the batch information distribution in the two-dimension plot of principle component 1 (PC1) and principle component 2 (PC2). The data adjusted by the *Combat* was then used for feature selection procedure in classification and differential methylation analysis. Feature selection was conducted by random forest and SVM with leave-one cross-validation. Differential methylation analysis was conducted by Wilcox signed-rank test without normality assumption. The most powerful panel was identified and the differential methylation status was estimated. In the validation stage, the methylation status of genes from the above panel (methylation genes combination) was detected in 150 NSCLC and normal tissues from the Chinese Han population by MSD-SNuPET. Logistic regression model, random forest, support vector machine (SVM), and Bayes tree were used to classify NSCLC in the validation data with fivefold cross-validation.

### Patients, samples and DNA

NSCLC samples and corresponding normal lung tissues for validation study in the Chinese population were obtained from 150 patients who underwent pulmonary resection for primary NSCLC at Changhai Hospital, Shanghai, China. The study was approved by Fudan University and Changhai Hospital, and informed consents were obtained from the patients. Exclusion criteria included subjects with a family history of lung cancer, previous radiotherapy, and chemotherapy or adjuvant therapy before surgery. All tissues were immediately frozen at -80°C after surgical resection. Histological examination and tumor-node-metastasis classification were conducted according to the World Health Organization classification criteria [31] and the AJCC Cancer Staging Manual, 7th Edition [32], respectively. Age, sex, smoking status, histology type, TNM stage and differentiation status were collected for use as the covariates when conducting the association between DNA methylation and disease status. Smoking status was assigned to a binary status: never and ever smoking. TNM stage was assigned to early stage (I and II) or late stage (III and IV) when necessary, so that the sample size can be big enough to get the efficient statistic power.

### Methylation status-dependent single nucleotide primer extension assay

DNA extraction and bisulfite conversion were performed as previously described [33,34]. Methylation status determined by the single nucleotide primer extension technique (MSD-SNuPET) was designed for the quantification of methylation at multiple methylated loci simultaneously. MSD-SNuPET was developed based on SNPshot technology to bisulfite converted CpG sites. An unmethylated cytosine would be converted to uracil when treated with bisulfite, whereas methylated cytosine maintains as the cytosine. Therefore, methylation status detection can be detected by specific primer and PCR amplification. Primer 3.0 was used to design primer sets (called the amplifying primers) which were applied to amplify genome regions including the target CpG sites. Allele-specific elongation primers were used to quantify the copy number of C and T alleles. Primer pairs were showed in Additional file 1: Table S2. PCR was performed in a final volume of 10 μL containing 1× HotStarTaq buffer, 3.0 mM Mg2+, 0.3 mM dNTP, 1 U HotStarTaq polymerase (Qiagen Inc. USA), 1 μl DNA template and 1 μl multiple primer set. Amplifications were conducted in a GeneAmp PCR System 9700 thermal cycler (Applied Biosystems, Foster City, CA) with the following thermal cycling profile: denaturation for 2 min at 95°C, followed by 11 cycles, each consisting of 20 sec at 94°C, 40 sec at 60°C, 90 sec at 72°C, and a final extension step for 2 min at 72°C. Negative and positive controls were included in each run of PCR as described above. The products of the sequencing reactions were purified and SNaPshot analysis of single nucleotides extension for multiple loci operation was shown as in our previous works [35]. DNA sequencing was conducted with the 3730 DNA analyzer. GeneMapper 4.1 (Applied Biosystems, Co., Ltd., USA) was used to analyze the fluorescence signals that represent different alleles. DNA methylation level was positively correlated with the magnitude of the C allele (*H*_*C*_) and negative corrected with the magnitude of the T allele (*H*_*T*_) in MSD-SNuPET technique (Additional file 1: Figure S1). In order to quantitatively estimate the methylation level for each CpG site, a standard calibration curve was established, in which synthetic DNA fragments of C and T alleles were mixed with C allele proportion at 10%, 20%, 30%, 35%, 40%, 50%, 60%, 70%, 75%, 80% and 90%, respectively. Then, a standard calibration curve could be fitted as a quadratic regression model: y = *β*_0_*x*^2^ + *β*_1_*x*, in which *β*_0_ and *β*_1_ are optimized parameters. *x* indicates the ratio of *H* and T alleles (*H*_*C*_/*H*_*T*_). In the present study, one technique and biological control were set. The reference site was a C site that was not in the CpG site; therefore, a low methylation signal should be detected and nonsignificant association should be detected between cancer and normal samples. Methylation status of *LINE-1* was taken as a biological control since we are clear that it is hypomethylation in the cancer tissues.

### Statistical analysis and machine learning

We selected methylated genes for classification by ranking genes with *P* values for testing differential methylation between tumor and normal tissue samples. We used three test statistics: student *t*-test, Wilcoxon rank sum test and Wilcoxon signed rank test statistic to test for differential methylation between two conditions for the normal distribution of methylation level, nonpaired tumor and normal tissue samples and paired tumor and normal tissue samples, respectively. False discovery rate (FDR) correction was used for multiple test correction with the R function of p.adjust with fdr as a parameter. Euclidean distance and partitioning around medoids were used to conduct hierarchical cluster analysis. Logistic regression (Package stats), support vector machine (SVM, Package e1071), random forest based classification (Package randomForest) and Bayes tree (Package BayesTree) were used to classify the NSCLC tumor and normal tissues. The optimized prediction model was built with the best prediction accuracy in the training dataset, and then, the sensitivity, specificity, accuracy were obtained from logistic regression, SVM, random forest and Bayes tree model in the test dataset with previous parameters applied in the training stage. All statistical analyses were conducted in R [36]. Protein-protein interaction networks were constructed by *String 9.0* to show the function network of the genes in our study [37].

## Additional file

Additional file 1:
**Supplementary Information for primer, dataset and principle of MSD-SNuPET.**

## Abbreviations

*AGTR1*: angiotensin II receptor, type 1
AUC: area under the curve
*GALR1*: galanin receptor 1
*LINE-1*: long interspersed element-1
MSD-SNuPET: methylation status determined single nucleotide primer extension technology
NSCLC: non-small cell lung cancer
*NTSR1*: neurotensin receptor 1
*SLC5A8*: solute carrier family 5, member 8
TCGA: The Cancer Genome Atlas Project
*ZMYND10*: zinc finger, MYND-type containing 10
