# Supplementary material for: Identification and validation of the methylation biomarkers of non-small cell lung cancer (NSCLC)
Source: Clin Epigenetics. 2015 Jan 22;7(1):3. doi: 10.1186/s13148-014-0035-3 (PMC4318209; doi:10.1186/s13148-014-0035-3)
Supplement: Additional file 1: — Supplementary Information for primer, dataset and principle of MSD-SNuPET. [file 13148_2014_35_MOESM1_ESM.docx]

## Additional file 1

Supplementary Table 1. Datasets used for meta-analysis

| Dataset | Acession | NSCLC | pNSCLC/normal | Jaurnal |
| --- | --- | --- | --- | --- |
| Karl T. Kelsey et al(2009)/USA | GSE16559 | 57 | 52 | Cancer Res |
| [Esteller M](http://www.ncbi.nlm.nih.gov/pubmed?term=%22Esteller%20M%22%5BAuthor%5D) et al(2012)/USA | GSE28094 | 33 | 3 | Genome Res |
| TCGA (2014)/USA | [TCGA](http://www.ncbi.nlm.nih.gov/geo/query/acc.cgi?acc=GSE35341) | 262 | 51 | TCGA |

TCGA (2014) represent the data were downloaded in 2014.

Supplementary Table 2. Datasets used for meta-analysis

|  | Forward Primer | Reverse Primer | Extension primer |
| --- | --- | --- | --- |
| *AGTR1* | GGTGAAYGTTGATTTGATAGTTG | CTCTATTTTACATTCCCTCCTCC | AAAAATAATACCACAATCRTCCC |
| *GALR1* | AAGGTATTAATGGATGAGGAGGT | CCACCTCCCCAATAAACTAAC | TTTTTTTTTTTTTTTTTTTTTTTGYGTATYGTGATTTTTAAGGGG |
| *NTSR1* | GGGTGAGYGTTTTAGGGAATTAG | CACCCAACTTCTAACACCCTAT | TTTTTTTTTTTTTTTTTTGGAATTAGGATTTYGAGYGGG |
| *SLC5A8* | TAYGTGAGGAATTGGAGTGGT | TTTATCCCACATTCCTCCAC | TTTTTTTTTTCTCATCTACTCAAATATCCCCRAC |
| *ZMYND10* | GGGTGGGGATGTTGTTATATT | AACTATCCTATCCCAAACTTTAACAC | TTTTTTTTTTTTTTTTTTTTTTTTGATGTTGTTATATTYGGGGAYGA |
| *LINE-1* | TTTGAGTTAGGTGTGGGATATAGTTT | AAAAAATAACGAACGCACCTAA | TTTTTTTTTTTTTTTTTTTTTTTAATCRAATCACTCCCACCC |

**
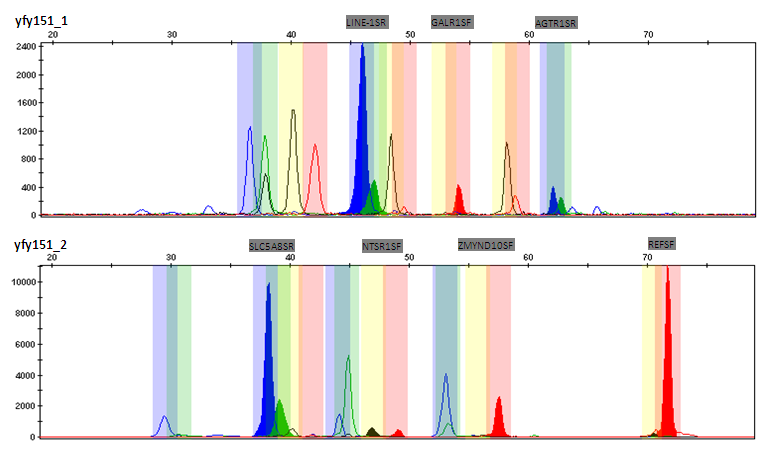
**

**
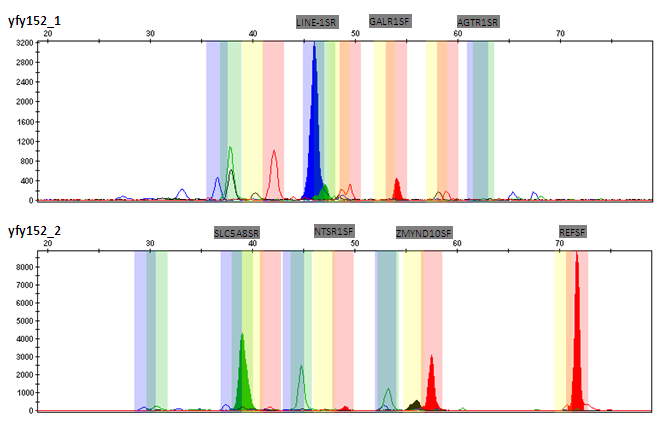
**

Supplementary Figure 1. Diagrammatic sketch of the result for multi-loci MSD-NEuTEP techonlogy

Length of the amplication production caused the fluorescence signals were showed in the different location for different genes when they are performed capillary electrophoresis. C or T allele could be determined by the color of the fluorescence signals. In this illustrated example.


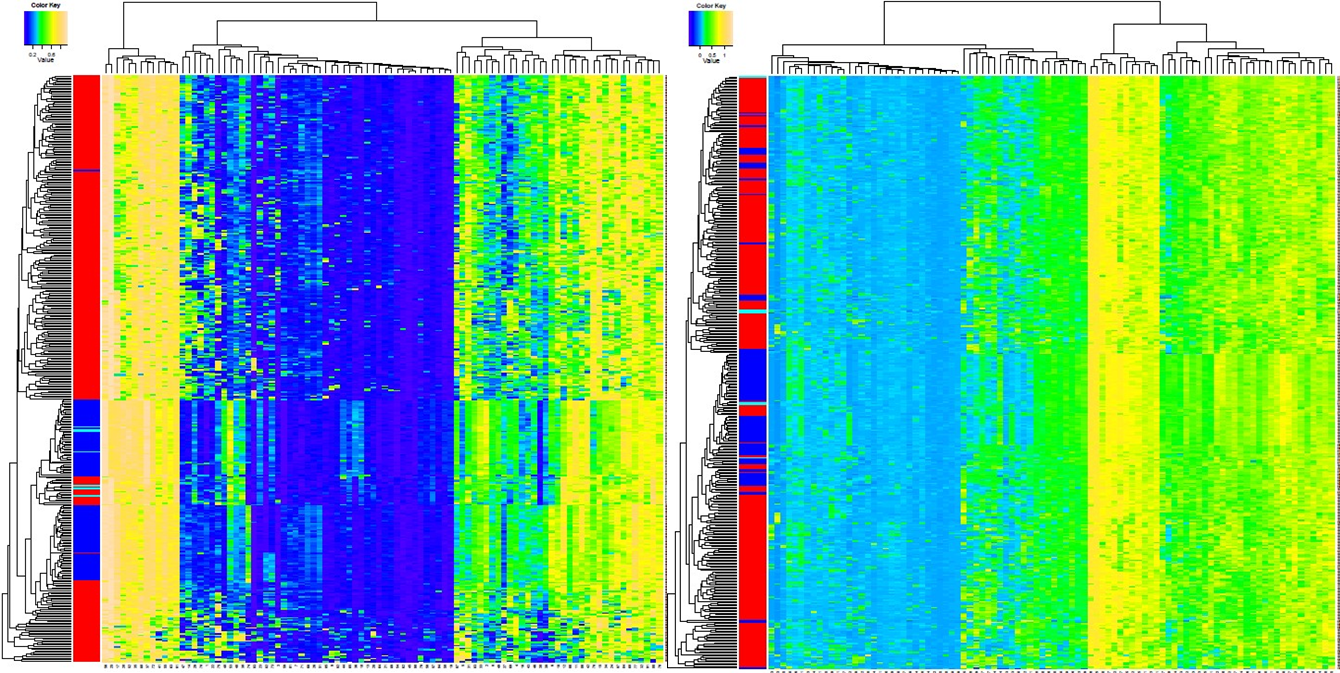


Supplementary Figure 2. Bilogical information could be preserved after Batch effect elimination.

Cluster analysis and heatmap plot were applied to show the data structure of the methylation profiling. A total of 120 probe sets with DNA methylation values after background and quantile normalization in 352 NSCLC and 106 normal samples were used in this analysis. X and Y axes represent the genes and samples, respectively. In the sample legends, red lines represent cancer tissues and green/blue represent adjacent/normal tissues.


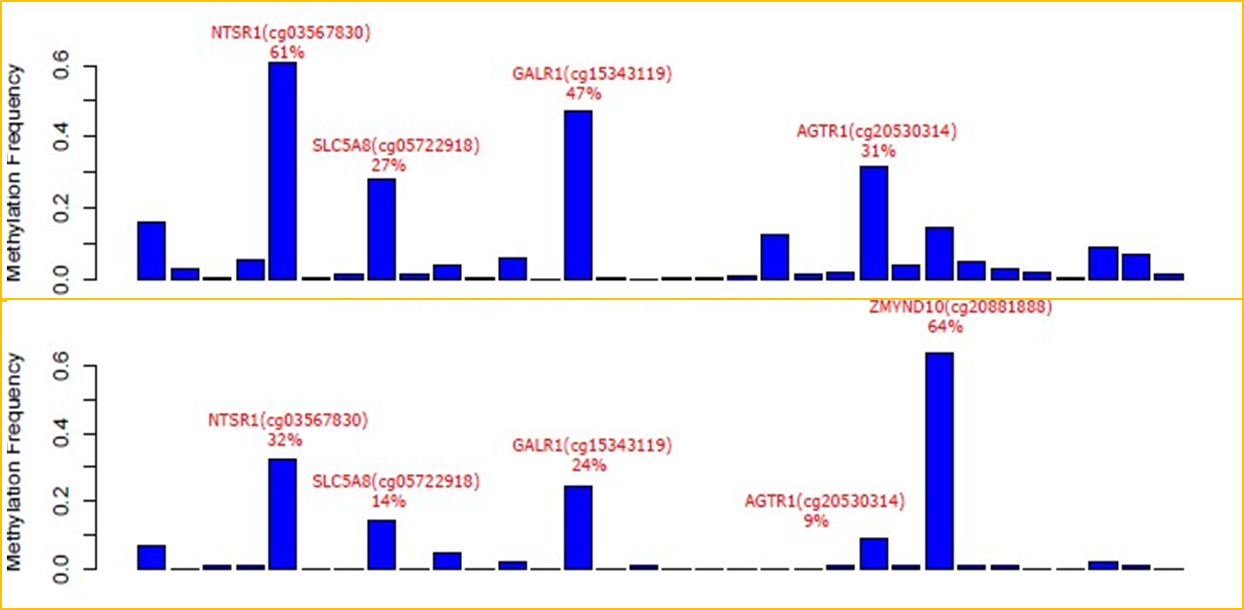


Supplementary Figure 2. DNA methylation frequency in NSCLC and normal tissues based on microarray meta-analysis

A total of 120 probes with DNA methylation values after background and quantile normalization in 352 NSCLC and 106 normal samples. Beta values < 0.3 defines the un-methylated CpGs and beta values > 0.8 the full methylated CpGs, beta values between 0.3-0.8 defines semi-methylated CpGs.


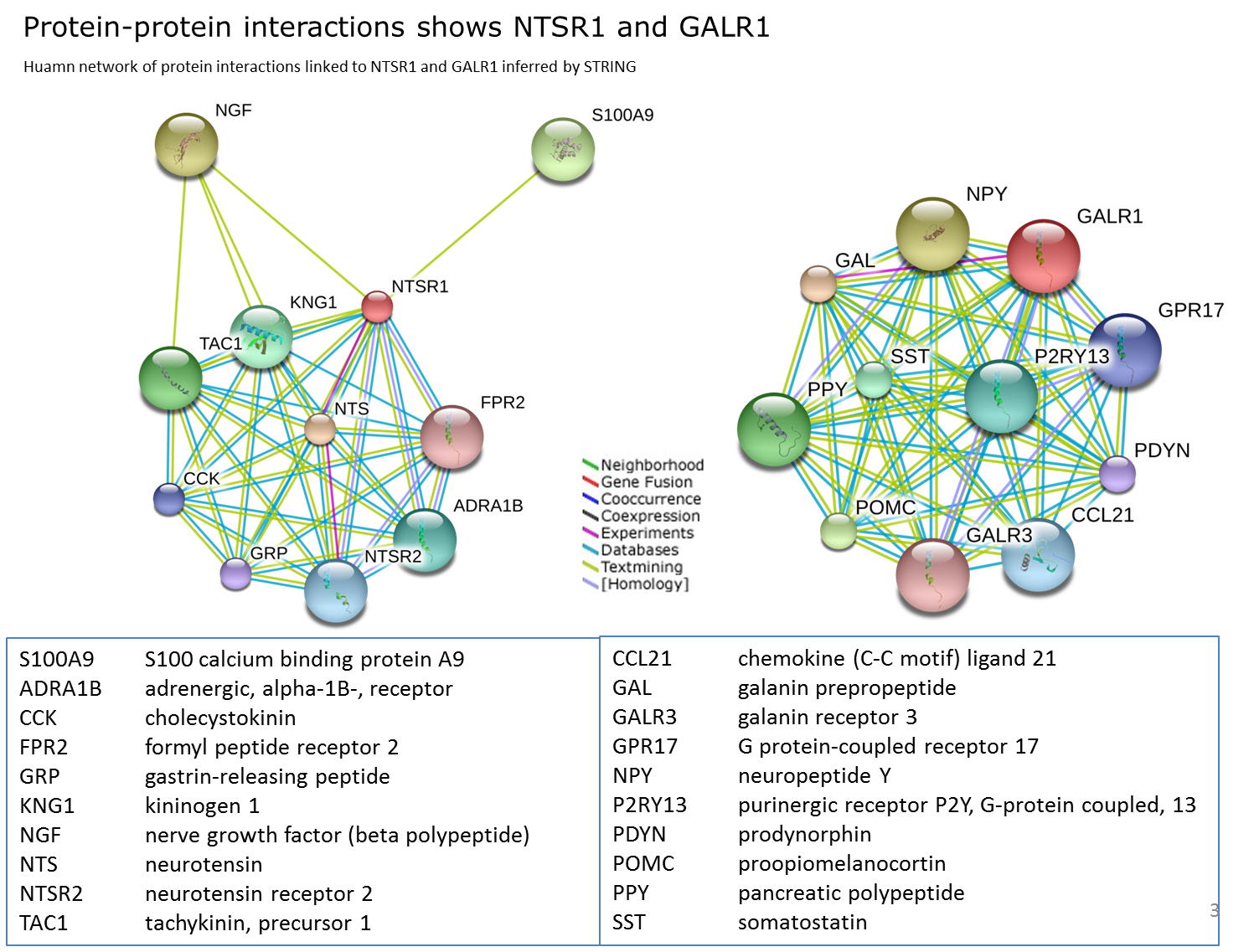


Supplementary Figure 4. Protein-protein interaction shows *NTSR1* and *GALR1*


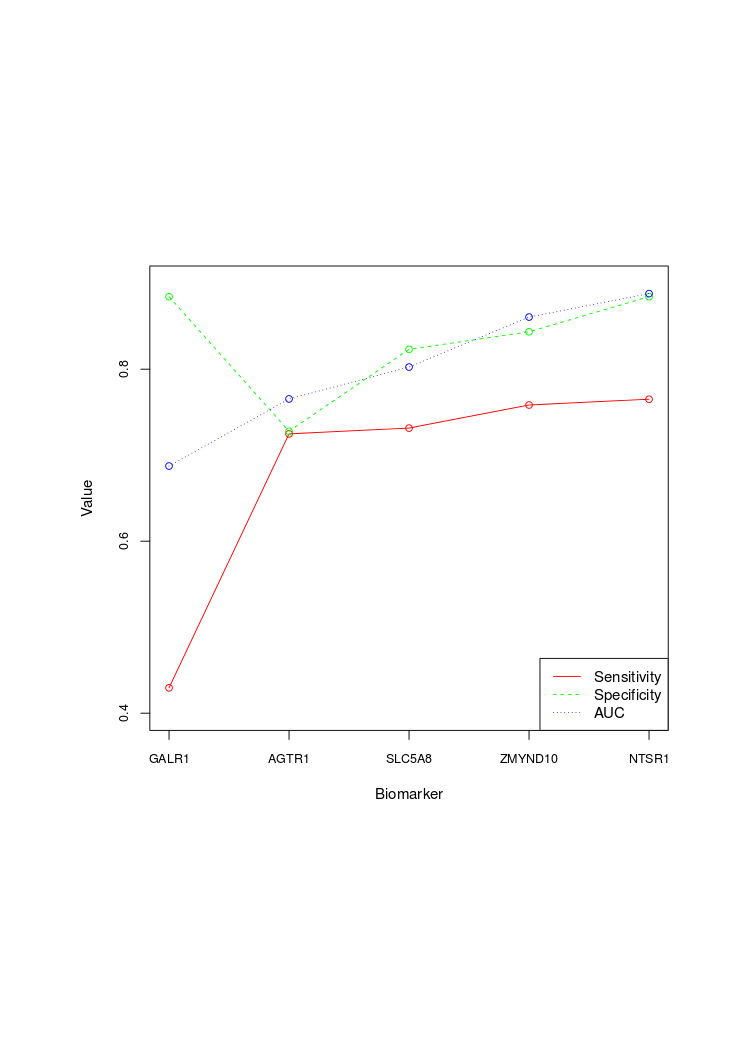


Supplementary Figure 5. Diagnostic performance of different panels of genes based on logistic regression model

Prediction baseline was set as gender, age and smoking. Each biomarker was added into the prediction model step by step according to the order provided by x-axis (that is GALR1, AGTR1, SLC5A8, ZMYND10 and NTSR1).
